# Proximal Biceps Tenodesis – Biomechanical Analysis in Sheep: Comparison between Metallic Anchor, Onlay Bioabsorbable Knotless Anchor, and Interference Screw

**DOI:** 10.1055/s-0043-1768616

**Published:** 2024-12-07

**Authors:** Amauri João Orso, Mateus Franceschi Dallanora, Paulo Cesar Faiad Piluski, Carlos Humberto Castillo Rodriguez, João Artur Bonadiman, Osvandré Lech

**Affiliations:** 1 Serviço de Ortopedia e Traumatologia, Hospital São Vicente de Paulo/Instituto de Ortopedia e Traumatologia, Passo Fundo, RS, Brasil

**Keywords:** biomechanical phenomena, models, animal, orthopedic fixation devices, tenodesis

## Abstract

**Objective** To biomechanically evaluate different fixation devices for the proximal biceps in the humerus of sheep, comparing their fixation strength to failure, tendon displacement, and failure site in each technique.

**Methods** A total of 27 humerus tests were performed on sheep, separating them into 3 groups: group A with tenodesis with metallic anchors ( *n* = 11), group B with biocomposite knotless devices ( *n* = 8) and group C with metallic interference screws ( *n* = 8), performing tenodesis with the sheep's own biceps, maintaining its native distal insertion. The three methods were submitted to a universal tensile testing machine.

**Results** There was no statistically significant difference in the strength of fixation until failure and displacement between the tendons fixed by the different techniques. Regarding the pattern of ruptures, it was observed that most ruptures of the metallic anchors occurred at the level of the myotendinous junction, most of the bioabsorbable knotless anchors failed due to slippage of the wire-screw interface, and all interference screws failed via tendon slip.

**Conclusion** The three techniques with metal anchor, onlay bioabsorbable knotless anchors, and interference screws are largely resistant to tensile loads for long head of the biceps tenodesis in sheep. There was no statistical difference between the three groups. Cyclic load resistance studies can provide more valuable data for comparing groups.

## Introduction

The anatomy of the brachial biceps is important for shoulder function. Proximally, it has two origins – one intra-articular and the other extra-articular. 1 Both bellies converge ∼ 7 cm proximal to the elbow, inserting themselves in the proximal radius. 2 3

The brachial biceps has the supination of the forearm as its primary function and, secondarily, acts as a flexor of the elbow. Proximally, its function has been constantly studied about its passive role in the superior stability of the glenohumeral joint. 3

Tendinopathies and ruptures of the proximal biceps are common sources of shoulder pain, making up 90% of all biceps lesions. 3 4 These conditions are usually related to previous tissue degeneration, having inflammatory, degenerative, traumatic and excessive use-related causes. 5 They have an important association with SLAP and rotator cuff pathologies, mainly subscapular tendon ruptures, which are closely related anatomically. 5

Decision-making regarding the treatment of biceps long-cord (BLC) pathologies may be conservative or surgical. Depending on the clinical presentation, provocative physical examinations, association with other shoulder pathologies and failure of conservative treatment. 2 3 5

In cases in which surgical treatment is indicated, several techniques are described, such as tenotomy and tenodesis, which may be open or arthroscopic. 4 5 6 7

The indication of tenodesis usually occurs in younger patients, athletes, and manual workers, and those who wish to avoid aesthetic deformities. Tenodesis allows the preservation of the length-tension ratio of the biceps, which can prevent postoperative muscle atrophy as well as feelings of fatigue cramps, helping to maintain the normal contour of the muscle. 2 3 4 5 6 The reinsertion site may also vary according to the profile and demand of the patient, the surgical technique employed, the underlying pathology, associated procedures, and surgeon preference. 1 2 3 4 5 6 7 8

There are several techniques described in the literature to perform BLC tenodesis, from the "rocambole" technique described by Godinho et al. 4 to techniques using fixation devices such as anchors, interference screw, and, more recently, bioabsorbable anchors without onlay nodes. 2 5 7 9 10 11 12

The present study aims to biomechanically evaluate the fixation of the BLC tendon in the humerus of sheep with bone metal anchors, bioabsorbable anchors without nodes, and interference screw, considering the resistance force, displacement of the tendon in relation to each device employed, and causes of failures of each technique.

## Materials and Methods

After approval by the ethics committee of the local educational institution, an experimental study was conducted in a biomechanics laboratory with 27 unfrozen fresh sheep humeri with a slaughter period of < 72 hours, aged between 8 and 12 months old. None of the samples appeared to have joint, bone, or tendon defect.

The weight of the pieces varied between 1,600 and 2,100 kg, with tests being performed at room temperature, with the samples taken from the cold chamber only for preparation of the parts. Afterwards, they were once again taken to the cold chamber at non-freezing temperature and tested with an interval of no more than 12 hours, to preserve the best quality of the piece.

**Fig. 1:**
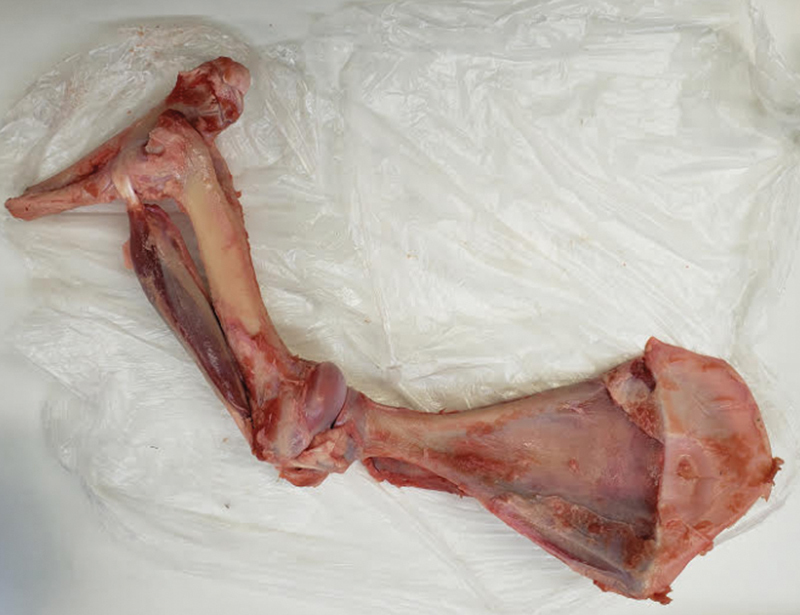
Anatomical part with native tendon, maintaining its distal origin and insertion.

It was chosen to use sheep shoulder joints because they reproduce an anatomy and bone density close, but not identical, to the human shoulder. 8 13 The shoulders were dissected evenly, keeping the humerus and distal insertion of the biceps with intact tendon for analysis. After dissection, BLC tenotomy was performed next to the supraglenoidal tubercle ( Fig. 1 ).

The fixation of the tenodesis in the bicipital drip was located 3 cm distal to the top of the large tuberosity. The Krakow-type stitch was used for the tenodesis technique.

The tenodeses were randomly divided into 3 groups: Group A with fixation with 5.5 mm metal anchor loaded with two high-strength Orthocord wires (Johnson & Johnson, Raynham, MA, USA), group B using the 5.5 × 20 mm SwiveLock (Arthrex, Naples, FL, USA) device, Group C with the 8 × 20 mm interference screw (Traumédica, Campinas, SP, Brazil).

**Fig. 2:**
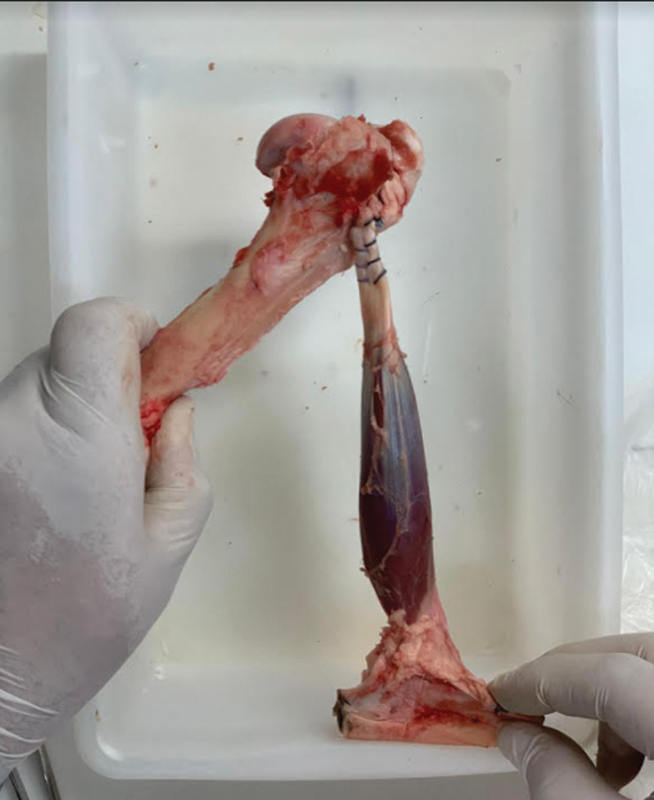
Fastening with metal anchor.

In group A, the fixation point was made with a specific initiator, obtaining a secure fixation to the humerus ( Fig. 2 ).

**Fig. 3:**
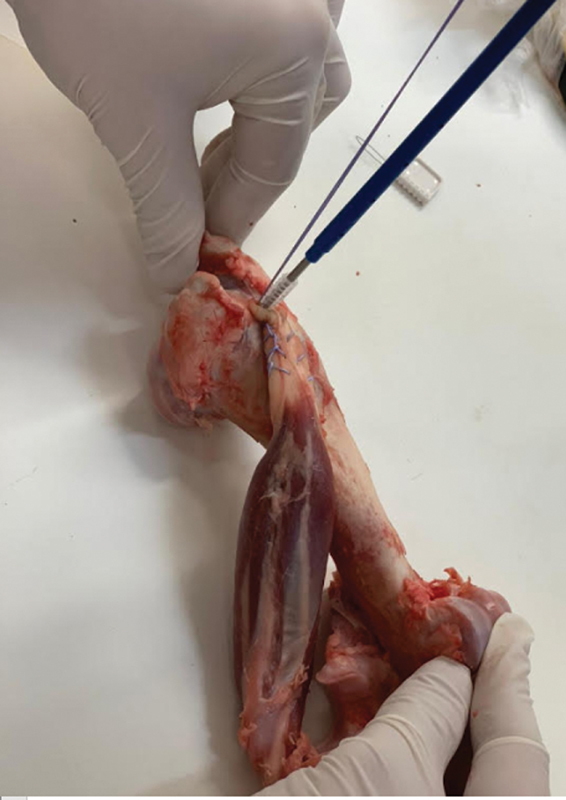
Anchor fixation without onlay bioabsorbable nodes.

For group B, the entry point was made with a specific initiator, and, after fixation, it was performed next to the tie with Krakow stitches to the tendon ( Fig. 3 ).

**Fig. 4:**
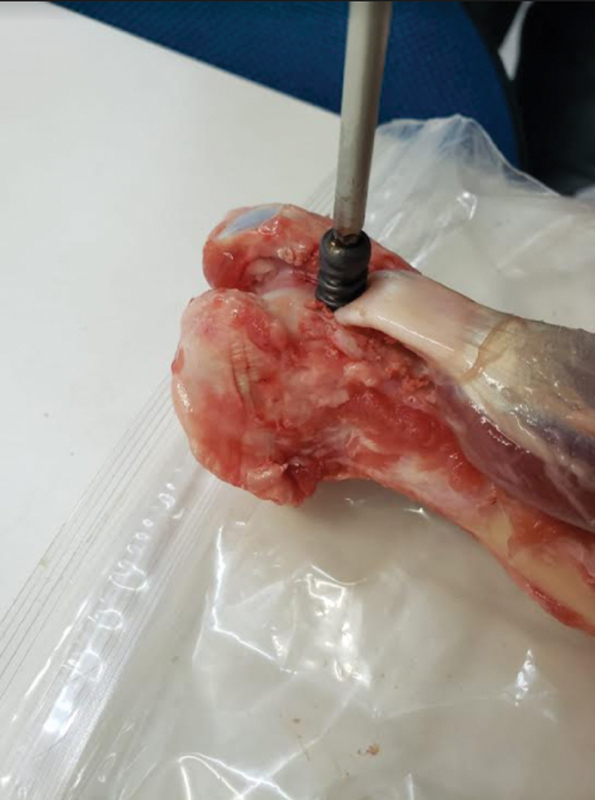
Tendon fixation with interference screw.

In group C, its entry point was performed with an adequate drill and, after fixation of the tendon, to the hole with the screw ( Fig. 4 ).

### Biomechanical Study

Biomechanical tests were applied using a universal continuous load machine until failure, with a speed of 20 mm/min. The force parameters were recorded through an 8-channel Spider data acquisition system (HBM, Darmstadt, Germany). The data processing software used was Catman Easy 3.1 (HBM, Darmstadt, Germany).

**Fig. 5:**
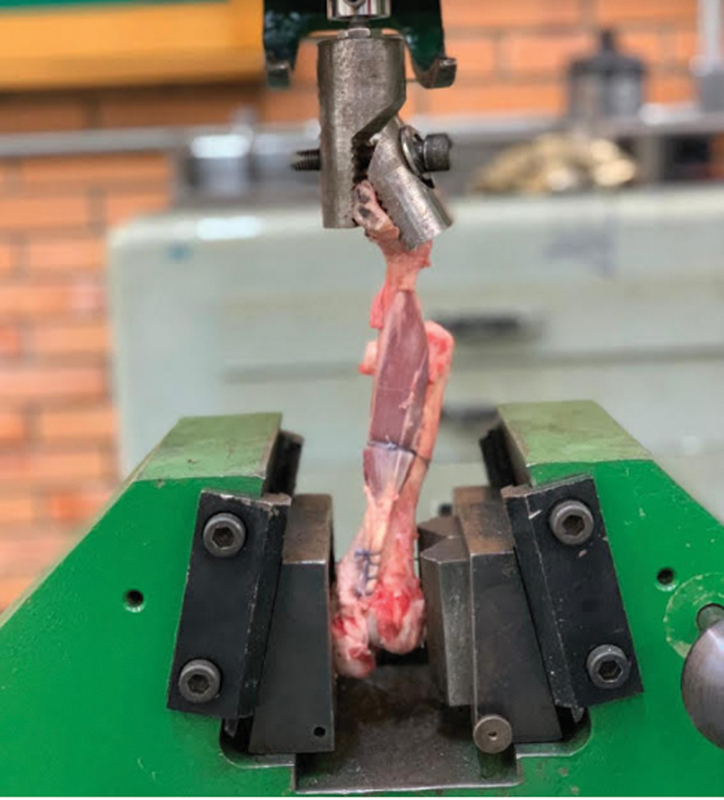
System mounted with metal anchor.

The humerus was fixed at the base by means of pressure on its articular surface and tuberosities. At the other point, the ulna and the radio were fixed by a metal clip, in a way that the tendon was completely free, and without tension, aligned on the axis with the anchoring device employed **,** maintaining an axial vector by the machine ( Fig. 5 ).

The parameters used for analysis were strength for resistance to failure of the system expressed in Newtons (N) and displacement in millimeters (mm), being established as system failure when there was a sudden drop in force during the test.

### Statistical Analysis

The data table was constructed using Microsoft Excel and statistical analysis was performed using IBM SPSS Statistics version 26 for Windows.

The numerical variables were expressed as mean ± standard deviation (SD) and categorical variables as absolute and relative frequency.

For peer-to-peer comparations, the *least significant difference* method was used, and the mean differences were expressed with the respective 95% confidence intervals.

Comparisons of rupture force and displacement with reference values were performed using variance analysis for repeated measurement, considering the difference between the observed value and the reference value as intra-subject effect and the fixation technique as a between-subject effect. The probability value was < 0.05.

## Results

**Fig. 6:**
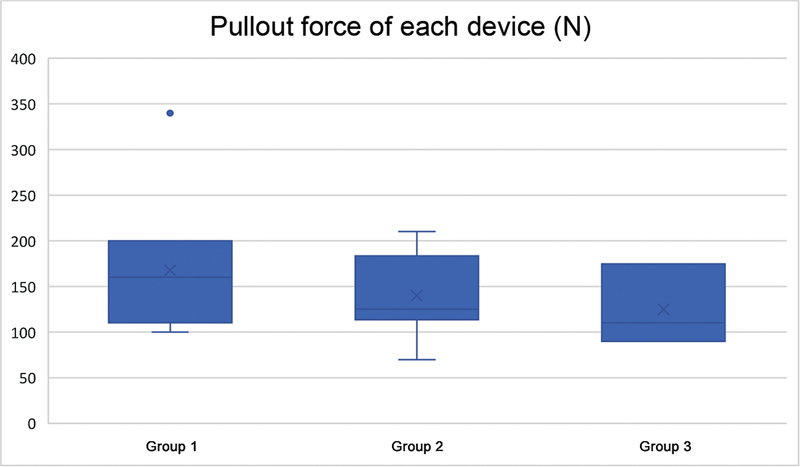
Boxplot presenting the pullout force of each device. Group 1: Metallic anchors ( *n* = 11), Group 2: Anchors without bioabsorbable nodes ( *n* = 8), Group 3: Interference screw ( *n* = 8).

There was no statistically significant difference in the rupture force between the tendons fixed by metallic anchor (167.7 ± 67.4 N), bioabsorbable anchor without nodes (140 ± 45.5 N), and interference screw (146.9 ± 73.3 N) ( Fig. 6 ).

It was observed that the displacement of tendons fixed with interference screw was significantly lower in relation to those fixed with bioabsorbable anchors without nodes, with a mean difference of 25.4 mm (95% confidence interval [CI]: 8.9 ± 42.0mm; *p* = 0.004). However, in the population in question, the displacement of the tendons fixed with interference screw was smaller than those fixed with metallic anchor; this difference was not statistically significant (mean difference: 14.1 mm; 95%CI: - 1.3 ± 29.5 mm; *p* = 0.072) ( Table 1 ).

**Table 1:** Comparison of the observed values for rupture and displacement force in the three different techniques evaluated ( *n* = 27)

	Technique			
	Metallic anchor	Anchor without bioabsorbable nodes	Interference screw	*p-value*
**Breaking force** (N)	167.7 ± 67.4	140.0 ± 45.5	146.9 ± 73.3	0.704
**Offset** (mm)	38.8 ± 18.7	50.2 ± 13.0	24.8 ± 14.8	0.015

Values expressed as mean ± standard deviation.

p: probability value.

Similarly, the difference in the displacement of tendons fixed with metallic anchor and bioabsorbable anchor without nodes was not statistically significant, having been on average 11.4 mm (4.0 ± 26.8 mm; *p* = 0.141).

Regarding the site of ruptures, in group A, of the 11 samples, 7 (63.6%) occurred at the level of the myotendinous junction, 3 (27.2%) at the Krakow suture level, and 1 (9.09%) case of anchor pullout. In group B, of the 8 samples, 7 (87.5%) resulted in failure with slipping of the wire fixation on the device, maintaining the high strength wire with tension until the final pullout of the device, while in 1 case (12.5%) there was failure with device breakage. In relation to group C, all samples had slipping of the screw-tendon interface (100%), as shown in Table 2 .

**Table 2:** Type of rupture

	Myotendinous	Slip fixing wire-device	Device pullout	Distal insertion rupture	Level breakage of the knot	Slip fixing tendon - device	Material breakage
Metallic Anchor ( *n* = 11)	5	0	1	2	3	0	0
Anchors without bioabsorbable nodes onlay ( *n* = 8)	0	7	0	0	0	0	1
Interference screw ( *n* = 8)	0	0	0	0	0	8	0

Regarding the maximum force until failure, the metal anchor supported more load (340 N) when compared with the bioabsorbable anchor without nodes (210 N) and the interference screws (330N) ( Table 3 ).

**Table 3:** Forces of devices in N

	Metallic anchor	Anchor without bioabsorbable nodes	Interference screw
Minor	100	110	25
Major	340	210	330

## Discussion

The present study compared the results of biomechanical linear tensile strength of the three fixation methods previously described.

It was observed that fixation with metallic anchor (167.7 ± 67.4 N) had greater fixation force than the bioabsorbable anchor without nodes (140 ± 45.5N) and than the interference screws (146.9 ± 73.3 N), but without statistical significance. Corroborating the article by Kilicoglu et al., 8 in which they evaluated 3 different biceps tenodesis techniques (soft tissue suture, anchors, and interference screw) in 45 shoulders of sheep *in vivo* , performing histological analysis at 0, 3, 6 and 9 weeks, demonstrating that the 3 groups had equivalent fixation strength at all times tested. 8

AlQahtani et al. 14 evaluated different options of BLC tenodesis and their clinical outcomes. In this review, the authors mention that BLC fixation is more commonly performed with the use of interference screws, due to its biomechanical superiorities regarding the pullout force of the screw and tendon. Although there was no statistically significant difference, our study showed that metal anchors have higher pullout resistance (167.7 ± 67.4 N) when compared with interference screws (146.9 ± 73.3 N). We can infer that this is because in fixation with the metallic anchor, there is a greater preservation of the cortical bone when compared with other devices that have a larger diameter, which could weaken the cortical components and reduce the force of the general fixation.

Regarding the mean values in relation to tensile strength, the analysis published by Ramos et al. 15 presented an average of 95 ± 35.3 N for bone anchors, 152.7 ± 52.7 N for interference screw, and 104.7 ± 23.54 N for soft tissue suture. Comparing with the data obtained in our study, we found very similar values when we analyzed the fixation method with interference screw (146.9 ± 73.3 N). However, when comparing the mean tensile strength with the method of fixation by metallic anchors, our study presented a significantly higher average (167.7 ± 67.4 N versus 95 ± 35.3 N). This variation of values can be attributed to the different patterns of anchors; for example, in this article, the metal anchor was loaded only with an Ethibond 2 wire, while in our investigation the anchor was loaded with two high-strength Orthocord wires. In the biomechanical study by Jayamoorthy et al., 16 the values obtained in the group with metallic interference screw were 210 ± 62 N, a considerably higher value compared with those obtained in our work with the interference screws (146.9 ± 73.3 N) and higher than that found by the analysis of Smuin et al. 17 , in which the mean value for bicep fixations per interference screw was 170.00 (±24.50 N). These variations can be attributed to the fact that the research by Jayamoorthy et al. 16 was performed on corpses while ours was carried out in sheep. In addition, the diameter of the interference screw used was 7 mm while in our research and in the publication of Smuin et al. 17 the screw with a diameter of 8 mm was used.

Regarding tendon displacement to failure, group C presented the lowest mean: 24.8 mm ± 14.8 mm. Group B, on the other hand, showed the highest displacement, with an average of 50.2 mm ± 13.0 mm. Comparing with the data we have in the literature, most studies that analyze this data evaluate the same based on cyclic loads 9 17 18 , which was not performed in our investigation. Biomechanical studies using anchors without bioabsorbable nodes for BLC tenodesis are still scarce. Lorbach et al. 18 performed a biomechanical evaluation comparing BLC fixation with conventional anchors and anchors without bioabsorbable SwiveLock nodes of 2 different diameters (5.5 and 8 mm). In this study, the 5.5 mm anchor presented a displacement after cyclic load greater than anchor fixation, which corroborates the findings in our study, in which, although we did not use cyclic load, the device with 5.5 mm diameter (Group B) was the one that presented the largest displacement until failure ( Table 1 ).

When analyzing the type of failure that occurred, it is observed that the variation occurred depending on the fixation method. In group A, among the 11 samples, only 1 case had device pullout (9.09%), while in group B, of the 8 samples, there was only 1 device break (12.5%) ( Table 2 ).

The seven failures due to myotendinous rupture of the three groups studied occurred in the longitudinal direction of the muscle fibers. Lopez-Vidriero et al. 19 observed a similar lesion in the longitudinal direction, but only in the tendon area, concluding that the quality of the tendon is important for this type of fixation.

There were three failures at the level of the surgical node in the metal anchor, due to its rupture. In these, the failure can happen due to fragility of the suture thread or even by the quality of the anchor that could have friction in the system. The eight failures of the interference screws occurred due to slipping of the tendon in relation to the screw, corroborating the findings in the literature. 15 16 17 18 19 20

Mazzoca et al. 20 performed an assay comparing four different proximal biceps tenodesis techniques (subpectoral biotenodesis interference screw, subpectoral bone tunnel technique, keyhole technique, or suture anchor) in a cyclic load in cadavers, demonstrating that there was no difference between resistance techniques until final failure, which is similar to the results of our analysis, in which all the techniques employed proved to be biomechanically similar and effective.

The present study used young ovine specimens at the point of slaughter to avoid maximum tendon, muscular and bone degeneration of the anatomical part. Although biomechanical investigations with animals (such as pigs, sheep, and cattle) are often found in the literature and can provide us with important comparative information 15 21 22 23 , we must consider that their applicability when compared to human bone is not equivalent. 24 25

Among the limitations that need to be mentioned in the present study, in addition to the nonequivalence between the bone structure of sheep and humans, our research evaluated the resistance of devices under a continuous, noncyclic, linear load, which would be more physiological and would be more like the reproduction performed i *n vivo* . Because we performed the analysis is *in vitro,* we do not consider the osteointegration factor of the implant to the bone, which occurs *in vivo.* We should also mention as a limitation the fact that we work with an *in vitro* tendon without any degeneration *,* since when performing the *in vivo* tenodesis we deal with a degenerate tendon.

## Conclusion

The three techniques: metallic anchor, no-knot anchor bioabsorbable onlay, and interference screw proved to be widely resistant to tensile loads for long biceps cable tenodesis in sheep. There was no statistically significant difference between the three groups. Studies with cyclic load resistance may provide more physiological data for group comparison.
