# Supplementary material for: Proximal Biceps Tenodesis – Biomechanical Analysis in Sheep: Comparison between Metallic Anchor, Onlay Bioabsorbable Knotless Anchor, and Interference Screw
Source: Rev Bras Ortop (Sao Paulo). 2024 Dec 7;59(5):e758–64. doi: 10.1055/s-0043-1768616 (PMC11624927; doi:10.1055/s-0043-1768616)
Supplement: Related texts

# Research article: Tenodese de bíceps proximal – Análise biomecânica em ovinos: Comparação entre âncora metálica, âncora sem nós bioabsorvível *onlay* e parafuso de interferência

**Keywords:** dispositivos de fixação ortopédica, fenômenos biomecânicos, modelos animais, tenodese

## Abstract

**Objetivo** Avaliar biomecanicamente diferentes dispositivos de fixação do bíceps proximal no úmero de ovinos, comparando sua força de fixação até a falha, deslocamento do tendão e local de falha em cada técnica.

**Métodos** Foram realizados 27 ensaios em úmero de ovinos, separando-os em 3 grupos: grupo A com tenodese com âncoras metálicas ( *n* = 11), grupo B com âncoras bioabsorvíveis sem nós ( *n* = 8) e grupo C com parafusos de interferência metálicos ( *n* = 8), realizando a tenodese com o próprio bíceps dos ovinos mantendo sua inserção distal nativa. Os três métodos foram submetidos a máquina universal de ensaio de tração.

**Resultados** Não se observou diferença estatisticamente significativa quanto à força de fixação até a falha e deslocamento entre os tendões fixados pelas diferentes técnicas. Em relação ao padrão de rupturas, observou-se que a maioria das rupturas das âncoras metálicas aconteceu ao nível da junção miotendínea, a maioria âncoras de suturas sem nós teve falha por escorregamento da interface fio-parafuso e todos os parafusos de interferência tiveram falha via escorregamento do tendão.

**Conclusão** As três técnicas com âncora metálica, âncora bioabsorvível sem nós e parafuso de interferência mostraram-se amplamente resistentes às cargas de tração para tenodese do cabo longo do bíceps em ovinos. Não houve diferença estatisticamente significativa entre os três grupos. Estudos com resistência em carga cíclica podem fornecer dados mais valiosos para a comparação dos grupos.

## Introdução

A anatomia do bíceps braquial é importante para a função do ombro. Proximalmente, o bíceps braquial possui duas origens – uma intra-articular e outra extra-articular. 1 Ambos os ventres se convergem cerca de 7 cm proximal ao cotovelo, inserindo-se no rádio proximal. 2 3

O bíceps braquial tem como função primária a supinação do antebraço e atua secundariamente como flexor do cotovelo. Proximalmente, sua função tem sido constantemente estudada em relação ao seu papel passivo na estabilidade superior da articulação glenoumeral. 3

Tendinopatias e rupturas do bíceps proximal são comuns fontes de dor no ombro, perfazendo 90% de todas as lesões do bíceps. 3 4 Essas condições geralmente estão relacionadas a uma prévia degeneração tecidual, tendo causas inflamatórias, degenerativas, traumáticas e relacionadas ao uso excessivo. 5 Apresentam importante associação com SLAP e patologias do manguito rotador, principalmente rupturas do tendão subescapular, com o qual está intimamente relacionado anatomicamente. 5

A tomada de decisão quanto ao tratamento das patologias do cabo longo do bíceps (CLB) pode ser conservadora ou cirúrgica, dependendo da apresentação clínica, de exames físicos provocativos, de associação com outras patologias do ombro e de falha do tratamento conservador. 2 3 5

Nos casos em que o tratamento cirúrgico é indicado, diversas técnicas são descritas, como tenotomia e tenodeses, podendo estas ser por via aberta ou artroscópica. 4 5 6 7

A indicação da tenodese geralmente ocorre em pacientes mais jovens, atletas e trabalhadores braçais, e aqueles que desejam evitar deformidades estéticas. A tenodese permite a preservação da relação comprimento-tensão do bíceps, o que pode prevenir a atrofia muscular pós-operatória bem como sensações de cãibras por fadiga, ajudando a manter o contorno normal do músculo. 2 3 4 5 6 O local de reinserção também pode variar de acordo com o perfil e a demanda do paciente, a técnica cirúrgica empregada, a patologia de base, procedimentos associados e preferência do cirurgião. 1 2 3 4 5 6 7 8

Existem diversas técnicas descritas na literatura para realização da tenodese do CLB, desde a técnica “rocambole” descrita por Godinho et al. 4 até técnicas utilizando dispositivos de fixação como âncoras, parafuso de interferência e, mais recentemente, âncoras bioabsorvíveis sem nós onlay. 2 5 7 9 10 11 12

O presente estudo tem por objetivo avaliar biomecanicamente a fixação do tendão do CLB no úmero de ovinos com âncoras metálicas ósseas, âncoras bioabsorvíveis sem nós e parafuso de interferência, considerando a força de resistência, o deslocamento do tendão em relação a cada dispositivo empregado e as causas de falhas de cada técnica.

## Materiais e Métodos

Após aprovação pelo comitê de ética da instituição de ensino local, foi realizado um estudo experimental em um laboratório de biomecânica com 27 úmeros de ovelhas frescas, não congeladas com período de abate < 72 horas, de idades entre 8 e 12 meses. Nenhuma das amostras aparentava defeito articular, ósseo ou tendíneo.

O peso das peças teve variação entre 1,600 e 2,100 Kg, com testes sendo realizados em temperatura ambiente, com as amostras retiradas da câmara fria somente para preparo das peças. Posteriormente, as amostras eram levadas novamente à câmara fria em temperatura não congelante e submetidas a testes com intervalo de no máximo 12 horas, visando preservar a melhor qualidade da peça.

Foi optado pelo uso de articulações de ombros ovinos por estes reproduzirem uma anatomia e densidade óssea próxima, porém não idêntica, à do ombro humano. 8 13 Os ombros foram dissecados uniformemente, mantendo-se para análise o úmero e a inserção distal do bíceps com tendão íntegro. Após a dissecção, era realizada a tenotomia do CLB junto ao tubérculo supra glenoidal ( Fig. 1 ).

**Fig. 1:** Peça anatômica com tendão nativo, mantendo origem e inserção distal.

A fixação da tenodese na goteira bicipital localizava-se 3 cm distal ao topo da grande tuberosidade **.** Para a técnica de tenodese, foi utilizado o ponto tipo Krakow.

As tenodeses foram aleatoriamente divididas em 3 grupos: o grupo A com fixação com âncora metálica de 5,5 mm carregada com 2 fios de alta resistência Orthocord (Johnson & Johnson, Raynham, MA, EUA), o grupo B com uso do dispositivo SwiveLock (Arthrex, Naples, FL, EUA) de 5,5 × 20 mm e o grupo C com uso do parafuso de interferência (Traumédica, Campinas, SP, Brasil) 8 × 20 mm.

No grupo A, o ponto de fixação era confeccionado com um iniciador específico, obtendo uma fixação segura ao úmero ( Fig. 2 ).

**Fig. 2:** Fixação com âncora metálica.

Para o grupo B, foi confeccionado o ponto de entrada com iniciador específico e, subsequentemente, foi realizada a fixação junto da amarria com pontos Krakow ao tendão ( Fig. 3 ).

**Fig. 3:** Fixação com âncora sem nós bioabsorvível onlay.

No grupo C, o ponto de entrada foi realizado com broca adequada e, subsequentemente, foi realizada a fixação do tendão ao orifício com o parafuso ( Fig. 4 ).

**Fig. 4:** Fixação do tendão com parafuso de interferência.

## Estudo Biomecânico

Testes biomecânicos foram aplicados utilizando uma máquina universal de carga contínua até falha, com velocidade de 20 mm/min. Os parâmetros de força foram registrados por meio de um sistema de aquisição de dados Spider (HBM, Darmstadt, Alemanha) com 8 canais. O software de processamento de dados utilizado foi o Catman Easy 3.1 (HBM, Darmstadt, Alemanha).

O úmero foi fixado na base por meio de pressão em sua superfície articular e tuberosidades. No outro ponto, a ulna e o rádio foram fixados por um clipe metálico, de uma maneira que o tendão ficasse totalmente livre e sem tensão, alinhado no eixo com o dispositivo de ancoragem empregado **,** mantendo um vetor axial pela máquina ( Fig. 5 ).

**Fig. 5:** Sistema montado com âncora metálica.

Os parâmetros utilizados para análise foram: força para resistência até falha do sistema expressa em Newtons (N) e deslocamento em milímetros (mm), sendo estabelecido como falha do sistema quando houvesse uma queda súbita de força durante o teste.

## Análise Estatística

A tabela de dados foi construída utilizando-se o Microsoft Excel (Microsoft Corporation, Redmond, WA, EUA) e a análise estatística foi realizada utilizando-se o IBM SPSS Statistics for Windows versão 26 (IBM Corp., Armonk, NY, EUA).

As variáveis numéricas foram expressas como média ± desvio padrão (DP) e as variáveis categóricas como frequência absoluta e relativa.

Para comparações aos pares, utilizou-se o método least significant diferences e as diferenças médias foram expressas com os respectivos intervalos de confiança (ICs) de 95%.

As comparações de força de ruptura e deslocamento com valores de referência foram realizadas utilizando-se análise de variância para medida repetida, considerando a diferença entre o valor observado e o valor de referência como efeito intra-sujeito e a técnica de fixação como efeito entre-sujeitos. Considerou-se como estatisticamente significativo valor de probabilidade < 0,05.

## Resultados

Não se observou diferença estatisticamente significativa quanto à força de ruptura entre os tendões fixados com âncora metálica (167,7 ± 67,4 N), com âncora bioabsorvível sem nós (140 ± 45,5 N) e com parafuso de interferência (146,9 ± 73,3 N) ( Fig. 6 ).

**Fig. 6:** Boxplot apresentando a força de arrancamento de cada dispositivo. Grupo 1: Âncoras Metálicas ( *n* = 11), Grupo 2: Âncoras Sem Nós Bioabsorvível ( *n* = 8), Grupo 3: Parafuso de Interferência ( *n* = 8).

Observou-se que o deslocamento dos tendões fixados com parafuso de interferência foi significativamente menor em relação aos fixados com âncoras bioabsorvível sem nós, com uma diferença média de 25,4 mm (IC95%: 8,9 ± 42,0 mm; *p* = 0,004). Apesar de, na população em questão, o deslocamento dos tendões fixados com parafuso de interferência ter sido menor que os daqueles fixados com âncora metálica, esta diferença não foi estatisticamente significativa, com uma diferença média de 14,1mm (IC95%: 1,3 ± 29,5 mm; *p* = 0,072) ( Tabela 1 ).

**Tabela 1:** Comparação dos valores observados para força de ruptura e deslocamento nas três diferentes técnicas avaliadas ( *n* = 27)

	Técnica			
	Âncora metálica	Âncora sem nós bioabsorvível	Parafuso de interferência	*valor-p*
**Força de ruptura** (N)	167,7 ± 67,4	140,0 ± 45,5	146,9 ± 73,3	0,704
**Deslocamento** (mm)	38,8 ± 18,7	50,2 ± 13,0	24,8 ± 14,8	0,015

Valores expressam média ± desvio padrão

Da mesma forma, a diferença do deslocamento dos tendões fixados com âncora metálica e com âncora bioabsorvível sem nós não foi estatisticamente significativa, tendo sido, em média, de 11,4mm (4,0 ± 26,8 mm; *p* = 0,141).

Em relação ao local das rupturas, no grupo A, das 11 amostras, 7 (63,6%) ocorreram ao nível da junção miotendínea, 3 (27,2%) ao nível do ponto Krakow e houve um (9,09%) caso de arrancamento da âncora. No grupo B, das 8 amostras, 7 (87,5%) resultaram em falha com escorregamento da fixação do fio no dispositivo, mantendo o fio de alta resistência com tensionamento até o arrancamento final dele, enquanto em um dos casos (12,5%) houve falha com quebra do dispositivo. Em relação ao grupo C, todas as amostras tiveram escorregamento da interface tendão-parafuso (100%), conforme exposto na Tabela 2 .

**Tabela 2:** Tipo de ruptura

	Miotendínea	Escorregamento fixação fio-dispositivo	Arrancamento do dispositivo	Ruptura inserção distal	Ruptura nível do nó	Escorregamento fixação tendão - dispositivo	Quebra do material
Âncora Metálica ( *n* = 11)	5	0	1	2	3	0	0
Âncoras sem nós bioabsorvíveis onlay ( *n* = 8)	0	7	0	0	0	0	1
Parafuso de interferência ( *n* = 8)	0	0	0	0	0	8	0

Em relação à força máxima até a falha, a âncora metálica suportou mais carga (340 N) quando comparada à âncora bioabsorvível sem nós (210 N) e os parafusos de interferência (330N) ( Tabela 3 ).

**Tabela 3:** Forças dos dispositivos em N

	Âncora metálica	Âncora sem nós bioabsorvível	Parafuso de interferência
Menor	100	110	25
Maior	340	210	330

## Discussão

O presente trabalho comparou os resultados de resistência de tração linear biomecânica dos três métodos de fixação previamente descritos.

Foi observado que a fixação com âncora metálica (167,7 ± 67,4 N) teve maior força de fixação do que a âncora bioabsorvível sem nós (140 ± 45,5N) e do que os parafusos de interferência (146,9 ± 73,3 N), porém sem significância estatística, corroborando o artigo de Kilicoglu et al., 8 que avaliaram 3 técnicas diferentes de tenodese do bíceps (sutura de partes moles, âncoras e parafuso de interferência) em 45 ombros de ovinos *in vivo* , realizando análise histológica em 0, 3, 6 e 9 semanas, demonstrando que os 3 grupos tiveram força de fixação equivalentes em todos os tempos testados. 8

AlQahtani et al. 14 avaliaram diferentes opções de tenodese do CLB e seus desfechos clínicos. Nessa revisão, os autores citam que a fixação do CLB é mais comumente realizada com o uso de parafusos de interferência devido às suas superioridades biomecânicas quanto à força de arrancamento do parafuso e tendão. Embora não tenha ocorrido diferença estatisticamente significativa, nosso estudo mostrou que as âncoras metálicas apresentam maior resistência de arrancamento (167,7 ± 67,4 N) quando comparadas com os parafusos de interferência (146,9 ± 73,3 N). Podemos inferir que isso se deve pelo fato de que, na fixação com a âncora metálica, ocorre uma preservação maior da cortical óssea, quando comparado com outros dispositivos que possuem maior diâmetro, os quais poderiam fragilizar os componentes corticais e reduzir a força da fixação geral.

Sobre os valores médios em relação à força de tração, a análise publicada por Ramos et al. 15 apresentou uma média de 95 ± 35,3 N para âncoras ósseas, 152,7 ± 52,7 N para parafuso de interferência e 104,7 ± 23,54 N para sutura em partes moles. Comparando com os dados obtidos em nosso estudo, encontramos valores muito semelhantes quando analisamos o método de fixação com parafuso de interferência (146,9 ± 73,3 N). Contudo, ao compararmos a força média de tração com o método de fixação por âncoras metálicas, nosso trabalho apresentou uma média significativamente maior (167,7 ± 67,4 N *versus* 95 ± 35,3 N). Essa variação de valores pode ser atribuída aos diferentes padrões das âncoras; por exemplo, no referido artigo, a âncora metálica era carregada apenas com um fio Ethibond 2, enquanto na nossa investigação a âncora era carregada com dois fios de alta resistência Orthocord. Já no estudo biomecânico de Jayamoorthy et al., 16 os valores obtidos no grupo com parafuso de interferência metálica foram de 210 ± 62 N, um valor consideravelmente maior em relação aos obtidos em nosso trabalho com os parafusos de interferência (146,9 ± 73,3 N), o qual também foi maior do que o encontrado pela análise de Smuin et al. 17 , onde o valor médio para as fixações do bíceps por parafuso de interferência foi de 170,00 ± 24.50 N. Essas variações podem ser atribuídas ao fato que a pesquisa de Jayamoorthy et al. 16 foi realizada em cadáveres enquanto o nosso foi realizado em ovinos. Além disso, o diâmetro do parafuso de interferência utilizado foi de 7 mm, enquanto na nossa pesquisa e na publicação de Smuin et al., 17 foi utilizado o parafuso com diâmetro de 8 mm.

Quanto ao deslocamento do tendão até a falha, o grupo C apresentou a menor média: 24,8 mm ± 14,8 mm. Já o grupo B foi o que apresentou maior deslocamento, com média de 50,2 mm ± 13,0 mm. Comparando com os dados de que dispomos na literatura, a maioria dos estudos que analisa este dado avalia o mesmo baseado em cargas cíclicas 9 17 18 , o que não foi realizado em nossa investigação. Ainda são escassos os estudos biomecânicos utilizando âncoras sem nós bioabsorvíveis para tenodese do CLB. Lorbach et al. 18 realizaram uma avaliação biomecânica comparando a fixação do CLB com âncoras convencionais e âncoras sem nós bioabsorvíveis SwiveLock de 2 diâmetros diferentes (5,5 e 8 mm). Neste estudo, a âncora de 5,5mm apresentou um deslocamento após carga cíclica maior do que a fixação com âncora, o que corrobora os achados em nosso trabalho, que, embora não tenha utilizado carga cíclica, o dispositivo com 5,5 mm de diâmetro (Grupo B) foi o que apresentou o maior deslocamento até a falha ( Tabela 1 ).

Ao analisar o tipo de falha ocorrida, observa-se que a variação ocorria dependendo do método de fixação. No grupo A, dentre as 11 amostras, em apenas 1 caso ocorreu arrancamento do dispositivo (9,09%). No grupo B, das 8 amostras, houve somente 1 quebra do dispositivo (12,5%) ( Tabela 2 ).

As sete falhas por ruptura miotendínea dos três grupos estudados ocorreram no sentido longitudinal das fibras musculares. Lopez-Vidriero et al. 19 observaram uma lesão parecida no sentido longitudinal, porém somente na área tendínea, concluindo que a qualidade do tendão é importante para este tipo de fixação.

Ocorreram três falhas ao nível do nó cirúrgico na âncora metálica, por ruptura da mesma. Nestes casos, a falha pode acontecer por fragilidade do fio de sutura ou mesmo pela qualidade da âncora que poderia ter atrito no sistema. As oito falhas dos parafusos de interferência aconteceram por escorregamento do tendão em relação ao parafuso, corroborando os achados na literatura. 15 16 17 18 19 20

Mazzoca et al. 20 realizaram um ensaio comparando quatro diferentes técnicas de tenodese de bíceps proximal (parafuso de interferência de biotenodese subpeitoral, técnica de túnel ósseo subpeitoral, técnica de keyhole, ou âncora de sutura) em aparelho com carga cíclica em cadáveres, demonstrando que não houve diferença entre técnicas quanto à resistência até a falha final, assim como nossa análise, na qual todas as técnicas empregadas mostraram-se biomecanicamente similares e eficazes.

O presente estudo utilizou espécimes ovinas jovens em ponto de abate, para evitar o máximo de degeneração tendínea, muscular e óssea da peça anatômica. Embora as investigações biomecânicas com animais (como suínos, ovinos e bovinos) sejam frequentemente encontradas na literatura e possam nos fornecer informações comparativas importantes 15 21 22 23 , devemos levar em conta que a sua aplicabilidade quando comparada ao osso humano não é equivalente. 24 25

Dentre as limitações que precisam ser mencionadas no presente estudo, além da já citada não-equivalência entre estrutura óssea dos ovinos e humanos, nossa pesquisa avaliou a resistência dos dispositivos sob uma carga linear contínua, e não cíclica, a qual seria mais fisiológica e se assemelharia mais à reprodução executada i *n vivo* . Pelo fato de a análise ser *in vitro,* não consideramos o fator osteointegração do implante ao osso, o que ocorre *in vivo.* Também devemos citar como limitação o fato de trabalharmos com um tendão *in vitro* sem degeneração alguma *,* visto que ao realizar a tenodese *in vivo* lidamos com um tendão degenerado.

## Conclusão

As três técnicas: âncora metálica, âncora sem nós bioabsorvível onlay e parafuso de interferência mostraram-se amplamente resistentes às cargas de tração para tenodese do CLB em ovinos. Não houve diferença estatisticamente significativa entre os três grupos. Estudos com resistência em carga cíclica podem fornecer dados mais fisiológicos para a comparação dos grupos.
